# Frequent and symmetric deposition of misfolded tau oligomers within presynaptic and postsynaptic terminals in Alzheimer’s disease

**DOI:** 10.1186/s40478-014-0146-2

**Published:** 2014-10-21

**Authors:** Hwan-Ching Tai, Bo Y Wang, Alberto Serrano-Pozo, Matthew P Frosch, Tara L Spires-Jones, Bradley T Hyman

**Affiliations:** 1 Department of Chemistry, National Taiwan University, 1 Roosevelt Road Section 4, Taipei, 106 Taiwan; 2 Department of Neurology, University of Iowa Hospitals & Clinics, Iowa City, IA USA; 3 C.S. Kubik Laboratory for Neuropathology, Massachusetts General Hospital, Harvard Medical School, Charlestown, MA USA; 4 Centre for Cognitive and Neural Systems and the Euan MacDonald Centre for Motor Neurone Disease, University of Edinburgh, Edinburgh, UK; 5 MassGeneral Institute for Neurodegenerative Disease, Massachusetts General Hospital, Harvard Medical School, Building 114, 16th Street, Charlestown, 02129 MA USA

## Abstract

The accumulation of neurofibrillary tangles in Alzheimer’s disease (AD) propagates with characteristic spatiotemporal patterns which follow brain network connections, implying trans-synaptic transmission of tauopathy. Since misfolded tau has been shown to transmit across synapses in AD animal models, we hypothesized that synapses in AD patients may contain misfolded tau. By immunofluorescence imaging of bipartite synapses from AD subjects, we detected tau protein in 38.4% of presynaptic and 50.9% of postsynaptic terminals. The pre/post distribution for hyperphosphorylated tau was 26.9%/30.7%, and for misfolded tau 18.3%/19.3%. In the temporal cortex, microscopic aggregates of tau, containing ultra-stable oligomers, were estimated to accumulate within trillions of synapses, outnumbering macroscopic tau aggregates such as tangles by 10000 fold. Non-demented elderly also showed considerable synaptic tau hyperphosphorylation and some misfolding, implicating the synapse as one of the first subcellular compartments affected by tauopathy. Misfolding of tau protein appeared to occur *in situ* inside synaptic terminals, without mislocalizing or mistrafficking. Misfolded tau at synapses may represent early signs of neuronal degeneration, mediators of synaptotoxicity, and anatomical substrates for transmitting tauopathy, but its actual role in these processes remain to be elucidated.

**Electronic supplementary material:**

The online version of this article (doi:10.1186/s40478-014-0146-2) contains supplementary material, which is available to authorized users.

## Introduction

The pathological hallmarks of Alzheimer’s disease (AD) are senile plaques and neurofibrillary tangles (NFT) [1],[2]. The accumulation of NFT, composed of misfolded, hyperphosphorylated tau proteins [3], follows a hierarchical spatiotemporal pattern which is consistent with anatomical connections in the brain [4],[5]. It therefore appears that NFT deposition spreads from one brain region to the next along major axonal projections, but the underlying mechanism remains unclear. Given the ability of misfolded tau to induce the misfolding of normal tau molecules via a seeding mechanism [6]-[10], it has been hypothesized that trans-synaptic transmission of misfolded tau molecules may underlie the spread of tauopathy [11]-[13], perhaps analogous to the spread of prion proteins within the brain [14]. Some have proposed that many neurodegenerative disorders may share the general feature of "prion-like" propagation of misfolded proteins [15],[16].

Studies in animal models have demonstrated that tauopathy can spread in the living brain, using either transgenic mice that express mutant human tau proteins specifically in the entorhinal cortex [11],[17], or by injecting tau aggregates into specific brain regions [18],[19]. However, these processes are relatively inefficient, taking weeks to months to observe, and occur only in situations of high levels of exogenous or transgenic tau. Tau has historically been identified as a microtubule-associated protein localized to the axon of mature neurons [20],[21], and a prerequisite for trans-synaptic propagation would be the localization of tau specifically at the synapse. We therefore hypothesized that, for the propagation model to be credible in human diseases, tau would need to be found at the synapse (at least in the disease state); if present at the synapse, the identification of tau species differentially present in pre- or post-synaptic elements, and in AD compared to controls, will test the further hypotheses that misfolded tau accumulates presynaptically before "release" into postsynaptic space, and that tau is mislocalized to the synapse in AD compared to normal neurons.

To test these hypotheses, we isolated and visualized intact, bipartite human neuronal synapses from cortical tissues of control and AD subjects. Using immunofluorescence to detect different forms of tau at bipartite synapses, we found normal tau protein to be symmetrically distributed across presynaptic and postsynaptic terminals in the normal human brain. Misfolded tau in AD-affected brains was also symmetrically distributed on both sides of the synapse, forming sodium dodecyl sulfate (SDS)-resistant oligomers. These data suggest that synaptic tau becomes hyperphosphorylated and misfolded *in situ* without significant spatial redistribution. Microscopic aggregates of misfolded tau deposited within synapses may represent early signs of neuronal degeneration, agents of synaptic toxicity, and anatomical substrates responsible for the transmission of tauopathy.

## Materials and methods

### Reagents

Protease inhibitor (cOmplete tablet) was purchased from Roche (Indianapolis, IN). Phosphatase inhibitor cocktails 2 and 3 were purchased from Sigma (St. Louis, MO) and used in 1:1 combination. High-quality Triton X-100 (glass ampule packaging) was purchased from Pierce (Rockford, IL). Mouse monoclonal antibodies DA9 (total tau), PHF1 (pS396/pS404 tau), and Alz-50 (misfolded tau) were gifts of Peter Davies (Albert Einstein College of Medicine). In accordance with original studies of Alz-50 antibody [22], we found Alz-50 to be weakly reactive against denatured tau in Western blotting after SDS-PAGE. So Alz-50 is a misfolded-conformation-specific tau antibody only under non-denaturing conditions, suitable for immunostaining of fixed cells/tissues.

Rabbit anti-tau (A20024) was purchased from Dako (Glostrup, Denmark); Rabbit anti-PSD95 (#2507) from Cell Signaling (Danvers, MA); Mouse anti-actin (A4700), rabbit anti-actin (A5060), and mouse anti-MAP2 (M4403) from Sigma; Mouse anti-synaptophysin (AB8049) from Abcam (Cambridge, MA); Rabbit anti-histone H3 (05-928) from Millipore (Billerica, MA); Mouse anti-GFAP (MS-1376) from Thermo (Waltham, MA).

### Human subjects

Brains from human subjects with a diagnosis of Alzheimer disease or no cognitive deficits were obtained through the Massachusetts Alzheimer’s Disease Research Center and Massachusetts General Hospital Neuropathology Department. All donor tissue was obtained in accord with local and national IRB regulations. Characteristics of control and AD subjects are listed in Table 1.

**Table 1:** **Characteristics of control and AD subjects examined in this study**

Case #	Age (y)	Gender	Diagnosis	Disease duration (y)	ApoE genotype	PMI (h)	Braak stage
C1	76	F	Control	NA	3/3	24	1
C2	80	F	Control	NA	2/4	54	1
C3	76	M	Control	NA	3/4	48	1
C4	89	F	Control	NA	2/3	13	2
C5	91	F	Control	NA	3/3	19	2
C6	71	M	Control	NA	NA	5	0
C7	87	M	Control	NA	NA	36	1
A1	85	F	AD	4	3/4	10	5
A2	73	F	AD	19	3/3	14	5
A3	84	F	AD	16	3/4	12	5
A4	92	M	AD	22	4/4	12	5
A5	83	F	AD	13	3/4	12	5
A6	82	M	AD	6	3/4	7	6
A7	91	F	AD	14	3/4	9	5
A8	95	M	AD	NA	3/3	11	6

NA = not applicable or not available; PMI = postmortem interval.

### Subcellular fractionation and protein extraction

Frozen human cortical tissue was dissected to separate grey matter from white matter, and 200-300 mg of thoroughly thawed grey matter was gently ground in a Potter-Elvehjem homogenizer with 1.5 mL ice-cold buffer A (25 mM HEPES pH 7.5, 120 mM NaCl, 5 mM KCl, 1 mM MgCl_2_, 2 mM CaCl_2_), supplemented with 2 mM DTT, protease inhibitors and phosphatase inhibitors. The homogenate was passed through two layers of 80 μm nylon filters (Millipore) to remove tissue debris, and a 200 μL aliquot was saved. The saved aliquot was mixed with 200 μL of water and 70 μL of 10% SDS, passed through a 27 gauge needle several times to shear DNA, and boiled for 5 min to prepare the total extract, followed by centrifugation at 15,000×g for 10 min to remove insoluble matter.

To prepare filtered synaptoneurosomes, the rest of the homogenate was passed through a 5 μm Supor membrane filter (PALL, Port Washington, NY) to remove large organelles and nuclei, and centrifuged at 1,000×g for 5 min to sediment synaptic terminals. Each pellet was resuspended in buffer A, split into two aliquots, and centrifuged again to yield two synaptoneurosome pellets. Supernatant from the first centrifugation step was clarified by centrifugation at 100,000×g for 1 h to obtain the cytosol fraction. Cytosolic extract was prepared by adding 1.5% SDS and boiling for 5 min. To prepare synaptoneurosome extracts, each synaptoneurosome pellet was mixed with 250 μL buffer B (50 mM Tris pH 7.5, 1.5% SDS, 2 mM DTT) and boiled for 5 min, followed by centrifugation at 15,000×g for 10 min to remove insoluble matter. Synaptoneurosome pellets may be snap frozen in liquid nitrogen and stored at -80°C.

### Immunostaining of synaptoneurosomes

Four-well Lab-Tek II CC2 (polyamine pre-coated) chamber slides (Nunc, Rochester, NY) were used for fixing and imaging synaptoneurosomes. Each synaptoneurosome pellet was resuspended in 5 mL of ice-cold buffer C (10 mM HEPES pH 7.9, 0.3 M sucrose) by gentle pipetting. Using a syringe with 27 gauge needle, 200 μL of synaptoneurosome suspension (containing about 25 μg of total protein) was transferred to each chamber well, followed by the addition of 200 μL 2% paraformaldehyde in ice-cold PBS-MC (phosphate buffered saline with 1 mM MgCl_2_ and 1 mM CaCl_2_). After 10 min of incubation with 1% paraformaldehyde at 4°C, synaptoneurosomes became fixed and crosslinked to the glass surface. Fixed synaptoneurosomes were washed with PBS-MC (room temperature from this point on) for three times (5 min each), and permeabilized for 10 min with 0.05% Triton X-100 in PBS-MC with 3% bovine serum albumin (BSA, Sigma), followed by three more washes. Slides were blocked with 4% normal goat serum (Invitrogen) and 3% BSA in PBS-MC for 30 min, and then incubated with primary antibodies diluted in PBS-MC with 3% BSA for 90 min, followed by three washes. Secondary antibodies diluted in PBS-MC with 3% BSA were added for 50 min, followed by three washes. The slide was mounted with #1.5 glass coverslip and Prolong Gold Antifade reagent (Invitrogen, Carlsbad, CA).

Primary antibodies for immunostaining included guinea pig anti-VGluT1 (Millipore AB5905, 1:150), chicken anti-MAP2 (Abcam AB5392, 1:100), DA9 (mouse IgG, 1:150), PHF1 (mouse IgG, 1:80), and Alz-50 (mouse IgM, 1:30). Fluorescent secondary donkey antibodies were purchased from Jackson Immunoresearch (West Grove, PA) and used at 1:100 dilutions (anti-guinea pig DyLight 649, anti-chicken Cy3, anti-mouse IgG Alexa 488, and anti-mouse IgM Alexa 488).

### Image acquisition and analysis

Immunofluorescence and brightfield images of synaptoneurosomes were acquired on an AxioImager Z1 epifluorescence microscope (Carl Zeiss, Oberkochen, Germany) equipped with a 63x oil immersion objective (N.A. = 1.40). Images were deconvolved with the Iterative Deconvolution plugin (by Bob Dougherty, OptiNav Inc.) in ImageJ software (version 1.44). This 2D deconvolution program required a point spread function (PSF) generated by Diffraction Limit PSF plugin (Bob Dougherty, OptiNav Inc.). For the brightfield image we used a 400 nm PSF; for green fluorescence channel a 509 nm PSF; for red channel a 550 nm PSF; for far red channel a 650 nm PSF. The optimal iteration number was empirically determined to be 12 for brightfield images and 16 for fluorescence images, with the LP filter diameter set at 1.5 pixels. After deconvolution, brightfield and fluorescence images were overlaid in ImageJ (using hyperstacks) and protein colocalization was determined by manual inspection. The cutoff threshold for fluorescence signals (after deconvolution) was generally set as two standard deviations above the mean. For stereological counting, we created a 12×8 grid image which was overlaid with microscope images, and devised a rule to randomly select different grid areas to look for synaptic terminals until the target number was reached. Two-way ANOVA was computed using Graphpad Prism software and t-tests were computed using Excel software.

### Classification of synapse morphology

During stereological counting, we defined a synaptic terminal as a brightfield object in the size range of 300-1000 nm with immunofluorescence signals for either presynaptic (VGlut1) or postsynaptic (MAP2) marker. Brightfield objects negative for both synaptic markers were regarded as non-synaptic organelles/vesicles, and these were not quantified, even if they were positively stained by tau antibodies. To qualify as a bipartite synapse, the brightfield object should show a snowman-like structure or a non-spherical shape being elongated or protruded, and pre/post makers needed to be non-overlapping (judged by the centers of mass of the puncta) and overlaid with the brightfield object in a reasonable manner to be considered as adjacent presynaptic and postsynaptic terminals. When synapses were clustered together with other objects under the brightfield, we did not attempt to classify them as bipartite or hemi synapses. There were also tau-positive puncta which did not overlap with brightfield objects and they were excluded from our analysis.

### Transmission electron microscopy

Synaptoneurosome pellets were fixed in 2% glutaraldehyde and 2% paraformaldehyde in PBS overnight at 4°C, rinsed, post-fixed in 1% osmium tetroxide, and embedded in LR White resin (Electron Microscopy Sciences, Hatfield, PA) according to manufacturer's protocols. Embedded blocks were cut into 70-nm thin sections on an Ultracut Microtome (Leica, Nussloch, Germany). Images were acquired on a JEOL1011 transmission electron microscope equipped with an ATM digital camera (JEOL USA, Peabody, MA).

### Gel electrophoresis and immunoblotting

SDS-denatured protein extracts were subjected to BCA assay (Pierce) to determine protein concentrations. Extracts were boiled again for 3 min after adding 5x sample buffer (250 mM Tris pH 7.5, 5% SDS, 400 mM DTT, 50% glycerol, 0.2% Orange G). Samples were resolved by SDS-PAGE using Bis-Tris 4-12% precast gels (Invitrogen), and transferred to low-fluorescence PVDF or nitrocellulose membranes (Millipore) for immunoblotting, detected using an Odyssey laser scanner (Li-Cor, Lincoln, NE). Blocking buffer and fluorescent secondary antibodies were purchased from Li-Cor and used according to manufacturer's protocols.

## Results

### Isolation and visualization of synaptic terminals from postmortem tissues

**Figure 1:**
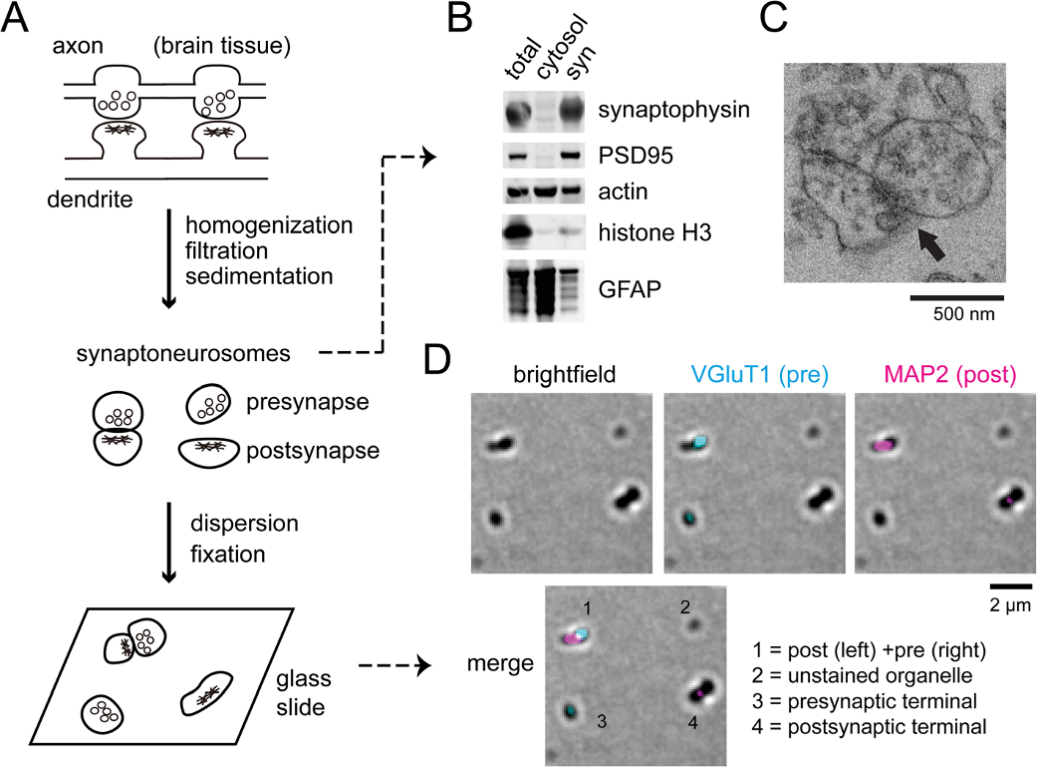
**Isolation of human neuronal synapses for optical imaging. (A)** Unfixed human brain tissue is homogenized, and synaptic terminals are collected by subcellular fractionation. Synapse-enriched fractions, called synaptoneurosomes, are sparsely fixed over glass slides for optical imaging. **(B)** Compared to total and cytosolic extracts, synaptoneurosomes (syn) analyzed by Western blotting show enrichment in presynaptic (synaptophysin) and postsynaptic (PSD95) markers, and reduction in nuclear (histone H3) and astrocyte (GFAP) markers. Actin is a loading control. **(C)** Transmission electron micrograph of synaptoneurosomes, with the arrow marking an intact synaptic cleft. **(D)** Light microscopy of synaptoneurosomes fixed over a glass slide. Synapses and organelles appear as dark objects in brightfield images. Immunostaining against VGluT1 and MAP2 marks presynaptic and postsynaptic terminals, respectively.

We recently developed a method for isolating human synaptic terminals from frozen (unfixed) postmortem brain tissues, based on homogenization, 80 and 5 μm filtration, and low-speed centrifugation (Figure 1A) [23]. The resulting subcellular fraction, called filtered synaptoneurosomes [24], was enriched in synaptic marker proteins (synaptophysin and PSD95) by immunoblotting (Figure 1B), and enriched in synaptic structures under transmission electron microscopy (Figure 1C, Additional file 1: Figure S1 and S2). Using a gentle formaldehyde fixation protocol to crosslink synaptoneurosomes onto polyamine-coated glass slides, we could visualize individual synaptic or organelle structures by brightfield microscopy (Figure 1D). Three channel immunofluorescence was utilized to detect the presence of tau (or its modified forms), the presynaptic marker (vesicular glutamate transporter 1, VGluT1), and the postsynaptic marker (microtubule-associated protein 2, MAP2).

A significant proportion of brightfield objects were negative for synaptic markers (like object 2 in Figure 1D), and by electron microscopy we observed non-synaptic organelles such as mitochondria and myelin in the synaptoneurosome preparation (Additional file 1: Figure S1). Synaptophysin/PSD95 are commonly used pre/post-synaptic markers, but we have previously observed that their respective puncta often showed significant spatial overlap [23], making it difficult to colocalize with a third protein. So instead we chose the VGlut1/MAP2 marker pair, which showed better puncta separation (see Figure 1D for instance). Presynaptic marker VGlut1 is expected to label the majority of glutamatergic presynaptic terminals in the cortex [25], and we have previously shown its usefulness for immunostaining human presynaptic terminals in array tomography experiments [26]. Although MAP2 is often used as a subcellular marker for dendrites, its distribution in dendritic spines has been clearly demonstrated by ultrastructural studies [27],[28]. In the absence of interfering signals from dendrites, we found it to be a useful marker for immunostaining postsynaptic terminals [23].

The enrichment of MAP2 in both mouse and human synaptoneurosomes was further demonstrated by immunoblotting with a different MAP2 antibody (mouse monoclonal, Additional file 1: Figure S3), apart from the chicken anti-MAP2 used for immunofluorescence. Low molecular weight MAP2 (<100 kDa) appeared to be more highly enriched in synaptoneurosomes than common synaptic markers synatophysin and PSD95 (Figure 1B), confirming the synaptic localization of MAP2. We have also tried phalloidin reagents to stain postsynaptic terminals but they worked poorly (data not shown) compared to anti-MAP2.

### Biochemical and immunocytochemical detection of synaptic tau misfolding

**Figure 2:**
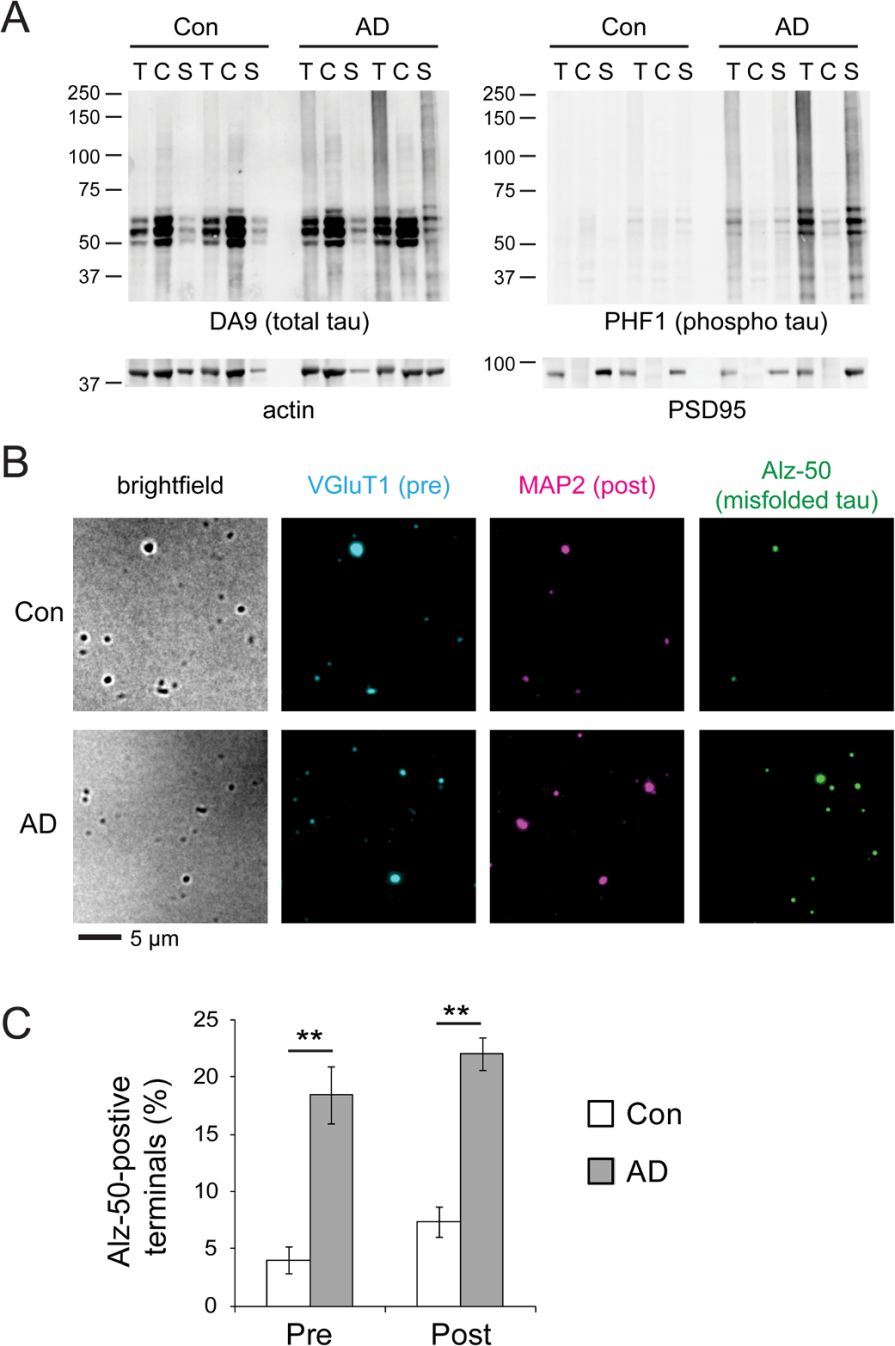
**Detection of misfolded tau in human synaptoneurosomes. (A)** SDS-PAGE and Western blots of total (T), cytosolic (C), and synaptoneurosomal (S) extracts show increases in hyperphosphorylated tau (detected by PHF1 antibody) and aggregated tau oligomers (smears above 75 kDa) in AD-affected synaptoneurosomes compared to non-demented controls. Actin and PSD95 are markers to confirm subcellular fractionation. **(B)** Using Alz-50 antibody to immunostain misfolded tau in synaptoneurosomes from control and AD subjects. VGluT1 and MAP2 are presynaptic and postsynaptic markers, respectively. **(C)** Quantification of Alz-50 immunostaining in synaptoneurosomes from control and AD subjects. Synaptic terminals of all morphologies were randomly counted based on stereology methods (3 controls, 300 presynapses, 195 postsynapses; 4 AD subjects, 400 presynapses, 320 postsynapses). Tau misfolding occurs more frequently in AD cases at both presynaptic and postsynaptic sites (two-tailed *t*-test, **p < 0.01).

Since misfolded tau proteins are generally believed to be seeds for transmitting proteinopathy [12],[13], we tried to detect their presence in synaptoneurosomes by two independent methods. The first method was SDS-polyacrylamide gel electrophoresis (PAGE) analysis, followed by immunoblotting (Figure 2A). In control cases, normal tau protein migrated as a monomer around 60 kDa, being more enriched in the cytosol (which included the axonal cytoplasm) than the synaptoneurosome, but with minimal phosphorylation. AD cytosolic extracts similarly contained mostly monomeric, non-phosphorylated tau. However, in AD synaptoneurosomes, tau became hyperphosphorylated and reactive against PHF1 antibody (pS396/pS404) [29], and misfolded into SDS-resistant oligomers that migrated as high molecular weight smears in the gel.

The second method utilized a conformation-specific antibody, Alz-50 [22], which recognized NFTs and dystrophic neurites in AD-affected brains [30],[31]. Immunostaining against Alz-50 was strongly elevated in synaptoneurosomes isolated from AD subjects compared to non-demented controls (Figure 2B). By stereological counting, we detected about 15% increase in Alz-50-positive terminals in AD subjects over controls (Figure 2C). Altogether, our data showed that tau underwent misfolding, hyperphosphorylation, and oligomerization at AD-affected synapses.

**Figure 3:**
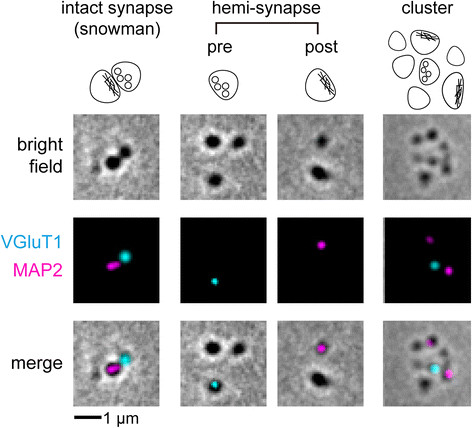
**Different morphologies of human synaptoneurosomes fixed over glass slides.** Intact, bipartite synapses appear as snowman-like or elongated/protruded structures under the brightfield, with VGluT1 (presynaptic marker) and MAP2 (postsynaptic marker) immunofluorescence on opposing sides. Isolated hemi-synapses and clusters of synapses are more abundant than bipartite structures. The ratio of bipartite synapse/hemi-synapse/clustered synapse is approximately 5%/50%/45%. Organelles negative for synaptic markers account for about half of the brightfield objects, and these were excluded from our analysis of tau immunofluorescence.

Stereological counting in Figure 2C was conducted over all synaptic terminals observable within chosen areas, but it could be potentially confounded by the uneven morphological distribution of synapses. So we further classified observed synapses into three common morphology types, as shown in Figure 3. The first morphology class was the intact, bipartite synapse, represented by snowman-like or elongated/protruded structures under brightfield, with immunostaining for VGluT1 and MAP2 on opposite sides. The second class was the isolated hemi-synapse, represented by a single object under brightfield, with immunostaining against either VGluT1 or MAP2. These represented detached presynaptic or postsynaptic (mushroom dendritic spine) terminals. The third class had multiple synaptic or non-synaptic structures clustered together (see Materials and methods section for detailed classification criteria).

**Figure 4:**
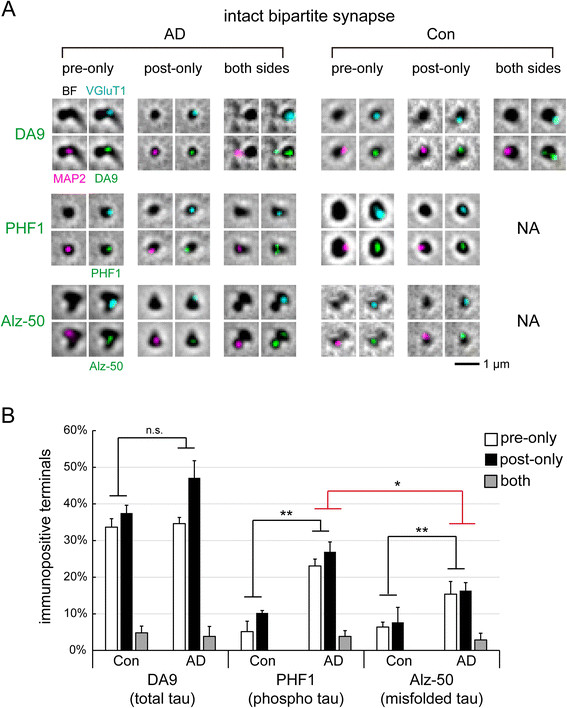
**Tau localization in bipartite synapses. (A)** Immunofluorescence detection by DA9 (total tau), PHF1 (pS396/pS404 tau), and Alz-50 (misfolded tau) in bipartite synapses, overlaid with brightfield (BF) images, from control and AD subjects. VGluT1/MAP2 are presynaptic/postsynaptic markers. NA = not available. **(B)** Quantification of tau immunofluorescence in bipartite synapses. Bilaterally positive synapses for PHF1 and Alz-50 were undetectable in control samples. 26 bipartite synapses per subject; 4 human subjects per group, except for control PHF1 and control Alz-50 experiments which had only 3 subjects. Statistical results (n.s. not significant, *p < 0.05, **p < 0.01) are shown for two-way ANOVA (pre/post vs. Con/AD, black line) and two-tailed *t*-test (red line).

**Figure 5:**
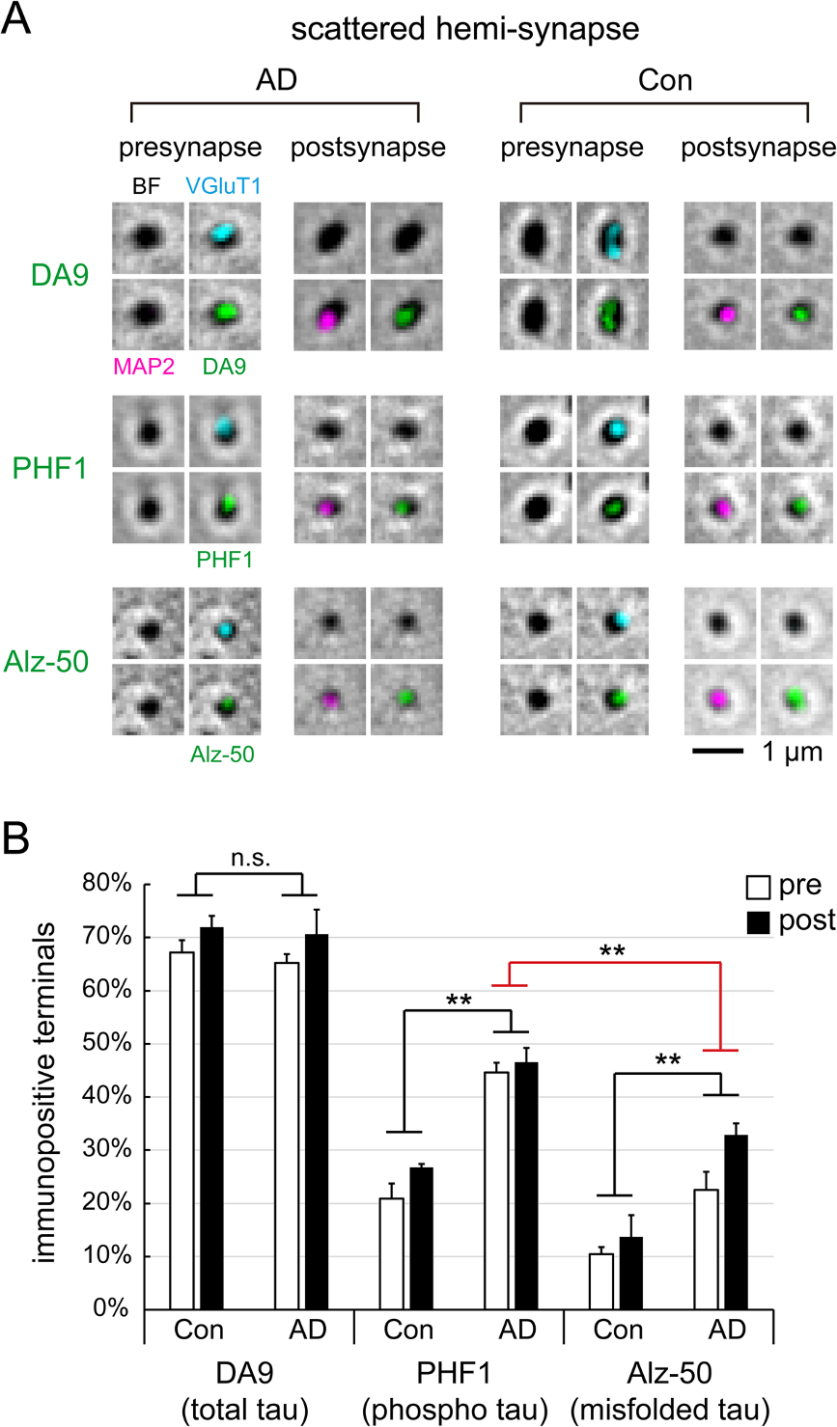
**Tau localization in hemi-synapses. (A)** Immunofluorescence detection by DA9 (total tau), PHF1 (pS396/pS404 tau), and Alz-50 (misfolded tau) in isolated hemi-synapses, overlaid with brightfield (BF) images, from control and AD subjects. VGluT1/MAP2 are presynaptic/postsynaptic markers. **(B)** Quantification of tau immunofluorescence in scattered hemi-synapses. 51 hemi-synapses per subject; 4 human subjects per group, except for control PHF1 and control Alz-50 experiments which had only 3 subjects. Statistical results (n.s. not significant, *p < 0.05, **p < 0.01) are shown for two-way ANOVA (pre/post vs. Con/AD, black line; pre/post vs. PHF1/Alz-50, red line).

To better understand the spatial distribution of tau in synaptic structures, we analyzed bipartite and hemi synapses separately (Figures 4B and 5B), in addition to summing over mixed morphologies (Figure 2C). In particular, bipartite synapses allowed us to unambiguously assign tau, or its phosphorylated or misfolded forms, into three distribution patterns: presynaptic-only, postsynaptic-only, or both sides.

### Tau localizes to synapses to a similar extent in normal aging and AD

In non-demented elderly, normal tau proteins were detected by DA9 antibody at 76.0 ± 2.9% (mean ± S.E.M.) of bipartite synapses, with a distribution ratio of 33.7%/37.5%/4.8% (pre-only/post-only/both) (Figure 4). It was surprising to find a slightly higher (not significant) fraction of postsynaptic terminals containing tau compared to presynaptic terminals, despite tau being concentrated in the axonal cytoplasm of mature neurons [20],[21].

We also examined isolated hemi-synapses from control subjects, finding tau inside 67.2 ± 2.3% of presynaptic terminals and 71.9 ± 2.1% of post synaptic terminals (Figure 5). By multiplying these two numbers, we would expect 44.0% of bipartite synapses to be tau-positive on both sides, if they arose from random associations of hemi-synapses. But the actual percentage of double-positive bipartite synapses was much lower, only 4.8 ± 1.8%, and the difference was highly significant (p < 10^-5^, two-tailed *t*-test). Altogether, our results clearly established that tau was abundantly distributed inside dendritic spines, in addition to being axonal and presynaptic.

DA9 antibody could recognize both phosphorylated and non-phosphorylated tau forms [32], and in AD samples 85.6 ± 5.1% of bipartite synapses were tau (DA9)-positive, with a distribution ratio of 34.6%:47.1%:3.8% (pre-only/post-only/both) (Figure 4B). It revealed that tau proteins did not mistraffic or mislocalize to synapses in AD, since they were abundantly present in synapses before AD.

### Abnormal tau forms increase in AD-affected synapses

In bipartite synapses of AD subjects (Figure 4B), immunostaining with Alz-50 antibody detected misfolded tau at both presynaptic and postsynaptic terminals, and the distribution ratio was 15.4%:16.4%:2.9% (presynaptic-only/postsynaptic-only/both), showing bilateral symmetry across the synaptic cleft. As such, tau misfolding occurred at 34.6 ± 5.7% (mean ± S.E.M.) of bipartite synapses from the temporal lobe, a region marked by high NFT burdens in AD [33]. The fraction containing hyperphosphorylated tau was even higher at 53.8 ± 4.7%, with a distribution ratio of 23.1%:26.9%:3.8% (pre-only/post-only/both). Tau hyperphosphorylation and misfolding were significantly higher in AD compared to non-demented controls (by two-way ANOVA, Table 2).

**Table 2:** **Summary of two-way ANOVA analyses of tau detection frequencies at bipartite synapses and hemi-synapses**

Tau detection type		Total tau		Phosphorylated tau		Misfolded tau	
Synapse type		Bipartite	Hemi	Bipartite	Hemi	Bipartite	Hemi
P value	Con vs. AD	n.s.	n.s.	0.0002	< 0.0001	0.0014	0.0004
				(Con < AD)	(Con < AD)	(Con < AD)	(Con < AD)
	pre vs. post	0.0236	n.s.	n.s.	n.s.	n.s.	0.0475
		(pre < post)					(pre < post)

n.s. = not significant.

By observing more frequent tau hyperphosphorylation than misfolding in bipartite synapses (p < 0.05, two-tailed *t*-test, Figure 4B), our data supported the observation that tau phosphorylation preceded fibril formation in the brain and in cellular models [1],[34],[35]. The probability of tau becoming hyperphosphorylated or misfolded was roughly equivalent at presynaptic and postsynaptic sites. This was also confirmed by counting hyperphosphorylated tau (PHF1) and misfolded tau (Alz-50) in isolated hemi-synapses from AD cases. The presynaptic:postsynaptic ratios were 44.6%:46.6% (phospho) and 22.6%:32.8% (misfolded), respectively (Figure 5B).

### Comparing control and AD synaptoneurosomes

The percentage of synapses (bipartite and hemi) positive for total tau was similar between control and AD subjects, but abnormal forms of tau was elevated in the latter (Figures 4B and 5B). However, appreciable amounts of abnormal tau were detectable in control synaptoneurosomes, with hyperphosphorylation found in 20.9%:26.8% (pre/post) and misfolding in 10.5%:13.7% (pre/post) of hemi-synapses (Figure 5B). It appeared that tau abnormality occurred at neocortical synapses in aging brains before apparent AD pathology (Braak stages 1 or 2). AD would lead to even more synaptic tau molecules being hyperphosphorylated and misfolded (Table 2), but the initial trigger might have occurred in the preclinical stage of AD.

All forms of tau examined in this study, normal or abnormal, generally showed similar frequencies on both sides of bipartite synapses. This was true in both control and AD subjects. Nor did we notice obvious intensity differences between presynaptic and postsynaptic signals after inspecting thousands of synaptic terminals, although we did not formally quantify fluorescence signals. The pre/post symmetry of tau distribution appeared to be an invariant feature of normal and AD-affected brains.

## Discussion

### Misfolded tau oligomers deposited at synapses have the potential to spread tauopathy

In this study, we detected misfolded tau in a significant fraction of presynaptic and postsynaptic terminals in AD subjects, which had two significant functional implications: (1) it could impair brain network function; (2) it could represent the anatomical substrate of tau synaptic transmission.

The "transmission theory" of AD neurofibrillary pathology [12],[13] predicts that there should be misfolded, seeding-capable tau molecules localized inside synaptic terminals. The true identity of tau species that seed intracellular/trans-cellular tau aggregation remains unknown in the human brain, but studies in cellular and animal models have suggested that they may be oligomers or protofibrils [36]-[39]. Recently, Lasagna-Reeves et al. [18] have immunopurified tau oligomers from AD brains that could propagate tauopathy in living mice, and such oligomers shared several similarities with synaptic tau oligomers found in this study: (1) they migrated as smears between 75-300 kDa in SDS-PAGE; (2) they were microscopic, amorphous aggregates instead of fibrillar.

Lasagna-Reeves et al. observed tau oligomers isolated from AD brains forming amorphous globular aggregates 4-8 nm in diameter [18], while Maeda et al. also reported granular tau oligomers from AD subjects in the range of 5-50 nm [40]. Although we could not resolve the size or number of microscopic aggregates within each synaptic terminal by optical microscopy, under electron microscopy we did not observe any fibril within synaptic structures (Additional file 1: Figure S2). It was likely that synaptic tau inclusions were also amorphous, granular oligomers smaller than 50 nm, which appeared to be the typical state of pre-fibrillar tau aggregates [41]. SDS-PAGE analysis showed that the core components of these aggregates were ultra-stable oligomers. We also observed that synaptic tau oligomers shared certain conformational features (recognized by Alz-50 antibody) with larger fibrillar inclusions like NFTs and neuropil threads [22],[30],[31]. Hence, it is conceivable that the former may be able to seed the formation of the latter.

### Tau is present at synapses in sufficient quantities to plausibly impact neural system function

The number of synapses and neurons in the human temporal cortex has been estimated to be around 40 trillion and 6 billion, respectively [42]. In temporal cortices of end-stage AD subjects, we estimate that several trillion synaptic terminals contain microscopic aggregates of misfolded tau. On the other hand, the number of NFTs was estimated to be a few hundred million in this brain region [33]. Given the extraordinary number of synapses carrying misfolded tau, which may easily reach many billions in a tauopathy-affected area, the spread of misfolded tau into interconnected brain regions may be quite appreciable even if the probability of transmission is exceedingly low, such as one millionth per day at each synapse.

Given the importance of synaptic plasticity in neural transmission and memory encoding, the frequent deposition of misfolded tau at synapses may contribute significantly to cognitive decline in AD. This was supported by our previous observation that tau oligomer levels, but not Aβ oligomer levels, correlated with the disruption of the ubiquitin-proteasome system at synapses [23]. Moreover, in a special group of non-demented elderly with AD-like neuropathology marked by high plaque and NFT burdens, we observed that they did not develop synaptic tau oligomers like AD subjects. In these cases, synaptic tau oligomers appeared to be better correlated with dementia than synaptic Aβ oligomers, NFTs, or plaques [43].

### Synaptic compartments may exhibit early signs of tauopathy

Although tau is highly enriched in axons as a microtubule binding protein, its presence in other cellular compartments such as somas, dendrites, and nuclei has been clearly documented in healthy rodent and monkey brains [44]-[46]. Tau also appears to interact with actin [47],[48] and various membranous structures within the neuron [49],[50], which may potentially explain its partitioning into postsynaptic terminals instead of just presynaptic terminals. With the progression of AD, misfolded tau accumulates in the somatodendritic compartment [30],[51], but we found that the percentage of pre- and post-synaptic terminals with total tau did not increase significantly with AD—only phosphorylation and misfolding did. We found all forms of tau to be symmetrically distributed across the synapse, with or without AD. These data argue against the spatial redistribution of synaptic tau molecules during AD pathogenesis, but instead, tau hyperphosphorylation and misfolding probably occurred *in situ* within each synaptic terminal.

Inside individual terminals, hyperphosphorylation probably preceded misfolding, because more synapses showed hyperphosphorylation than misfolding in AD brains. In non-demented elderly, tau hyperphosphorylation was observation in ~20% of hemi-synapses (Figure 5B), which may be an early sign of tau abnormality. We do not know if this process began at a much younger age, or if it was the result of abnormal tau being synaptically transmitted from tangle-bearing regions. Most elderly over 65 would be characterized as Braak Stage 1 or 2, with tangles already present in the transentorhinal region [4],[30].

Hyperphosphorylation is known to precede macroscopic tau aggregation during AD pathogenesis [1],[30], but the subcellular origin of tau abnormality remains unclear. Our data suggest that one of the early signs of tau abnormality may be hyperphosphorylation inside synaptic terminals, even in non-demented elderly. Hyperphosphorylated tau loses its ability to bind microtubules [34],[52],[53], thereby gaining the tendency to misfold.

### Possible directionality in synaptic tau transmission

Recent studies using tauopathy mouse models have shown that normal and misfolded tau both transmit across brain regions via synaptic connectivity instead of spatial proximity [11],[17],[19],[54]. Moreover, both anterograde and retrograde spreading of tauopathy have been observed along axonal tracts, suggesting that tauopathy also transmits bidirectionally at synapses [19]. Our data appear to be consistent with the possibility of bidirectional transmission, since misfolded tau is equally likely to be presynaptic or postsynaptic within a bipartite synapse, but infrequently on both sides.

**Figure 6:**
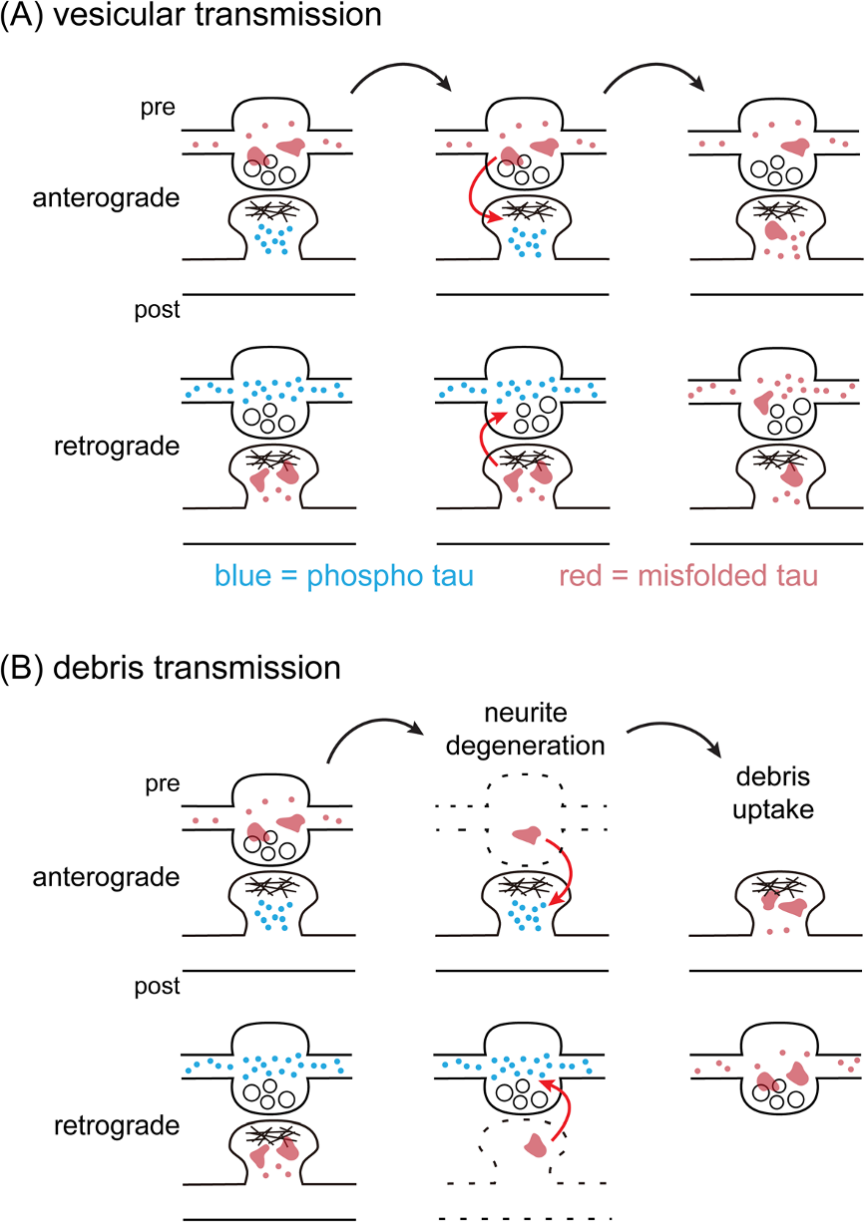
**Two plausible transmission mechanisms of misfolded tau across synapses.** Having hyperphosphorylated tau (blue dots) and misfolded tau (red dots) at opposing sides of the synapse may create an opportunity for spreading tauopathy. The infectious species, likely to be oligomers (red irregular shapes), may either transmit by **(A)** vesicular trafficking, or **(B)** debris uptake. In (B), as tauopathy damages a neuron to inflict neurite degeneration, tau oligomers may be incompletely degraded and remain as debris in the extracellular space. The neighboring synaptic terminal may accidentally endocytose the debris containing tau oligomers, seeding further tau misfolding.

Recent studies have identified tau in secreted vesicles of neuronal and non-neuronal cells [49],[55], and in the vesicle fraction of cerebrospinal fluids of early AD patients [56]. Others have shown that membrane-free tau aggregates added to culture media could also enter neurons to induce further aggregation [36],[37],[39],[57]. Hence, there are two possible routes for misfolded tau to cross into another neuron via synaptic contacts, which are illustrated in Figure 6.

The first pathway (Figure 6A) involves vesicular transport between presynaptic and postsynaptic terminals [58]. The symmetric distribution of misfolded tau we observed suggests that either direction of transmission seems plausible. The second model (Figure 6B) is prompted by the astronomical number of synaptic tau aggregates observed. As a tauopathy-stricken neuron dies, what happens to the hundreds of microscopic tau inclusions inside its synapses, especially in distal neurites? Micro-aggregates containing SDS-resistant tau oligomers are unlikely to be completely removed by the proteolytic systems of a failing neuron. Some aggregates may remain in its original place after the neurite disintegrates, much like a ghost tangle that stands in the place of a disintegrated neuron. It is conceivable that oligomeric tau debris may be taken up by neighboring neurites/synapses, causing tauopathy to spread in both retrograde and anterograde directions.

Had we observed misfolded tau depositing at both sides of the bipartite synapse with higher probability than chance, it might have provided indirect evidence for the vesicular transmission model in Figure 6A. This was one of the original goals of our experimental design. However, in our synaptoneurosome preparation, only a few percent of bipartite synapses contained tau protein on both sides, much lower than the expected value (Figure 4B), and the underlying reason remains unclear. Since well-characterized bipartite synapses were also rare in our preparation, the sample number was too small for estimating transmission probability. Further studies will be required to provide supporting evidence for the models proposed above.

## Conclusion

In this study we developed a new procedure to image intact synapses isolated from postmortem brain tissues and conducted the first quantitative assessment of synaptic tauopathy in AD. We demonstrated that tau misfolding occurs symmetrically across the synapse with high frequencies of oligomer deposition, revealing for the first time the extraordinary number of synaptic terminals containing microscopic tau inclusions. This methodology would be equally applicable to other neurodegenerative tauopathies such as progressive supranuclear palsy, certain forms of frontotemporal dementia, or corticobasal degeneration [59]. The underlying reason that AD, but not other tauopathies [51],[60], shows a hierarchical pattern of NFT deposition originating in transentorhinal regions still remains unanswered. Comparing the synaptic distribution of tau and analyzing its biochemical state in different tauopathies may provide insights into their respective pathogenesis mechanisms. The discovery of misfolded tau oligomers inside synaptic terminals highlights novel targets for pathogenesis studies and therapeutic interventions. For instance, tau immunotherapy may be able to sequester extracellular misfolded tau after it escapes from pre- or post-synaptic compartments but before it enters another neuron. It remains to be seen if synaptic deposition of misfolded proteins is a common mechanism of neurodegenerative proteinopathies, and imaging of isolated human synapses will be very useful for such investigations.

## Authors’ contributions

HCT carried out staining and biochemical experiments and drafted the manuscript. BYW conducted image and statistical analysis. AS and MPF contributed to case selection and tissue preparation. TLS carried out electron microscopy. BTH conceived the study, designed the experiments, and revised the manuscript. All authors discussed and approved the manuscript.

## Additional file

## Electronic supplementary material

Additional file 1: Figure S1: Transmission electron micrographs of human synaptoneurosomes isolated from non-demented controls, with arrowheads marking the presence of (1) myelin fragment; (2) intact synapse; (3) mitochondrion. **Figure S2:** Transmission electron micrographs of synaptoneurosomes isolated from Alzheimer’s disease subjects. No visible fibrils were observed. **Figure S3:** Immunoblotting with mouse anti-MAP2 and mouse anti-synaptophysin against total and synaptoneurosome (syn) extracts from human controls and mouse brain tissues. Low molecular weight MAP2 (<100 kDa) is highly enriched inside both human and mouse neuronal synapses. (PDF 739 KB)

Below are the links to the authors’ original submitted files for images.

Authors’ original file for figure 1

Authors’ original file for figure 2

Authors’ original file for figure 3

Authors’ original file for figure 4

Authors’ original file for figure 5

Authors’ original file for figure 6

## References

[1] Braak E, Griffing K, Arai K, Bohl J, Bratzke H, Braak H (1999). Neuropathology of Alzheimer’s disease: what is new since A. Alzheimer?. Eur Arch Psychiatry Clin Neurosci.

[2] Serrano-Pozo A, Frosch MP, Masliah E, Hyman BT (2011) Neuropathological alterations in Alzheimer disease. Cold Spring Harb Perspect Med 1:a006189 10.1101/cshperspect.a006189 PMC3234452 22229116

[3] Wood JG, Mirra SS, Pollock NJ, Binder LI (1986). Neurofibrillary tangles of Alzheimer disease share antigenic determinants with the axonal microtubule-associated protein tau. Proc Natl Acad Sci U S A.

[4] Braak H, Braak E (1991). Neuropathological stageing of Alzheimer-related changes. Acta Neuropathol.

[5] Arnold SE, Hyman BT, Flory J, Damasio AR, Van Hoesen GW (1991). The topographical and neuroanatomical distribution of neurofibrillary tangles and neuritic plaques in the cerebral cortex of patients with Alzheimer’s disease. Cereb Cortex.

[6] Friedhoff P, von Bergen M, Mandelkow EM, Davies P, Mandelkow E (1998). A nucleated assembly mechanism of Alzheimer paired helical filaments. Proc Natl Acad Sci U S A.

[7] Montejo de Garcini E, Serrano L, Avila J (1986). Self assembly of microtubule associated protein tau into filaments resembling those found in Alzheimer disease. Biochem Biophys Res Commun.

[8] Morozova OA, March ZM, Robinson AS, Colby DW (2013). Conformational features of tau fibrils from Alzheimer’s disease brain are faithfully propagated by unmodified recombinant protein. Biochemistry.

[9] Patterson KR, Remmers C, Fu Y, Brooker S, Kanaan NM, Vana L, Ward S, Reyes JF, Philibert K, Glucksman MJ, Binder LI (2011). Characterization of prefibrillar Tau oligomers in vitro and in Alzheimer disease. J Biol Chem.

[10] Rankin CA, Sun Q, Gamblin TC (2007) Tau phosphorylation by GSK-3beta promotes tangle-like filament morphology. Mol Neurodegener 2:12 10.1186/1750-1326-2-12 PMC1936422 17598919

[11] de Calignon A, Polydoro M, Suarez-Calvet M, William C, Adamowicz DH, Kopeikina KJ, Pitstick R, Sahara N, Ashe KH, Carlson GA, Spires-Jones TL, Hyman BT (2012). Propagation of tau pathology in a model of early Alzheimer’s disease. Neuron.

[12] Mohamed NV, Herrou T, Plouffe V, Piperno N, Leclerc N (2013). Spreading of tau pathology in Alzheimer’s disease by cell-to-cell transmission. Eur J Neurosci.

[13] Walker LC, Diamond MI, Duff KE, Hyman BT (2013). Mechanisms of protein seeding in neurodegenerative diseases. JAMA Neurol.

[14] Colby DW, Prusiner SB (2011) Prions. Cold Spring Harb Perspect Biol 3:a006833 10.1101/cshperspect.a006833 PMC3003464 21421910

[15] Frost B, Diamond MI (2010). Prion-like mechanisms in neurodegenerative diseases. Nat Rev Neurosci.

[16] Jucker M, Walker LC (2013). Self-propagation of pathogenic protein aggregates in neurodegenerative diseases. Nature.

[17] Liu L, Drouet V, Wu JW, Witter MP, Small SA, Clelland C, Duff K (2012) Trans-synaptic spread of tau pathology in vivo. PLoS One 7:e31302 10.1371/journal.pone.0031302 PMC3270029 22312444

[18] Lasagna-Reeves CA, Castillo-Carranza DL, Sengupta U, Guerrero-Munoz MJ, Kiritoshi T, Neugebauer V, Jackson GR, Kayed R (2012) Alzheimer brain-derived tau oligomers propagate pathology from endogenous tau. Sci Rep 2:700 10.1038/srep00700 PMC3463004 23050084

[19] Ahmed Z, Cooper J, Murray TK, Garn K, McNaughton E, Clarke H, Parhizkar S, Ward MA, Cavallini A, Jackson S, Bose S, Clavaguera F, Tolnay M, Lavenir I, Goedert M, Hutton ML, O'Neill MJ (2014). A novel in vivo model of tau propagation with rapid and progressive neurofibrillary tangle pathology: the pattern of spread is determined by connectivity, not proximity. Acta Neuropathol.

[20] Binder LI, Frankfurter A, Rebhun LI (1985). The distribution of tau in the mammalian central nervous system. J Cell Biol.

[21] Kosik KS, Finch EA (1987). MAP2 and tau segregate into dendritic and axonal domains after the elaboration of morphologically distinct neurites: an immunocytochemical study of cultured rat cerebrum. J Neurosci.

[22] Wolozin B, Davies P (1987). Alzheimer-related neuronal protein A68: specificity and distribution. Ann Neurol.

[23] Tai HC, Serrano-Pozo A, Hashimoto T, Frosch MP, Spires-Jones TL, Hyman BT (2012). The synaptic accumulation of hyperphosphorylated tau oligomers in Alzheimer disease is associated with dysfunction of the ubiquitin-proteasome system. Am J Pathol.

[24] Hollingsworth EB, McNeal ET, Burton JL, Williams RJ, Daly JW, Creveling CR (1985). Biochemical characterization of a filtered synaptoneurosome preparation from guinea pig cerebral cortex: cyclic adenosine 3':5'-monophosphate-generating systems, receptors, and enzymes. J Neurosci.

[25] Micheva KD, Busse B, Weiler NC, O’Rourke N, Smith SJ (2010). Single-synapse analysis of a diverse synapse population: proteomic imaging methods and markers. Neuron.

[26] Kay KR, Smith C, Wright AK, Serrano-Pozo A, Pooler AM, Koffie R, Bastin ME, Bak TH, Abrahams S, Kopeikina KJ, McGuone D, Frosch MP, Gillingwater TH, Hyman BT, Spires-Jones TL (2013). Studying synapses in human brain with array tomography and electron microscopy. Nat Protoc.

[27] Caceres A, Binder LI, Payne MR, Bender P, Rebhun L, Steward O (1984). Differential subcellular localization of tubulin and the microtubule-associated protein MAP2 in brain tissue as revealed by immunocytochemistry with monoclonal hybridoma antibodies. J Neurosci.

[28] Morales M, Fifkova E (1989). Distribution of MAP2 in dendritic spines and its colocalization with actin. An immunogold electron-microscope study. Cell Tissue Res.

[29] Greenberg SG, Davies P, Schein JD, Binder LI (1992). Hydrofluoric acid-treated tau PHF proteins display the same biochemical properties as normal tau. J Biol Chem.

[30] Braak E, Braak H, Mandelkow EM (1994). A sequence of cytoskeleton changes related to the formation of neurofibrillary tangles and neuropil threads. Acta Neuropathol.

[31] Hyman BT, Van Hoesen GW, Wolozin BL, Davies P, Kromer LJ, Damasio AR (1988). Alz-50 antibody recognizes Alzheimer-related neuronal changes. Ann Neurol.

[32] Rojo LE, Alzate-Morales J, Saavedra IN, Davies P, Maccioni RB (2010). Selective interaction of lansoprazole and astemizole with tau polymers: potential new clinical use in diagnosis of Alzheimer’s disease. J Alzheimers Dis.

[33] Gomez-Isla T, Hollister R, West H, Mui S, Growdon JH, Petersen RC, Parisi JE, Hyman BT (1997). Neuronal loss correlates with but exceeds neurofibrillary tangles in Alzheimer’s disease. Ann Neurol.

[34] Mandelkow EM, Mandelkow E (2012) Biochemistry and cell biology of tau protein in neurofibrillary degeneration. Cold Spring Harb Perspect Med 2:a006247 10.1101/cshperspect.a006247 PMC3385935 22762014

[35] Bancher C, Brunner C, Lassmann H, Budka H, Jellinger K, Wiche G, Seitelberger F, Grundke-Iqbal I, Iqbal K, Wisniewski HM (1989). Accumulation of abnormally phosphorylated tau precedes the formation of neurofibrillary tangles in Alzheimer’s disease. Brain Res.

[36] Frost B, Jacks RL, Diamond MI (2009). Propagation of tau misfolding from the outside to the inside of a cell. J Biol Chem.

[37] Guo JL, Lee VM (2011). Seeding of normal Tau by pathological Tau conformers drives pathogenesis of Alzheimer-like tangles. J Biol Chem.

[38] Nonaka T, Watanabe ST, Iwatsubo T, Hasegawa M (2010). Seeded aggregation and toxicity of α-synuclein and tau: cellular models of neurodegenerative diseases. J Biol Chem.

[39] Wu JW, Herman M, Liu L, Simoes S, Acker CM, Figueroa H, Steinberg JI, Margittai M, Kayed R, Zurzolo C, Di Paolo G, Duff KE (2013). Small misfolded Tau species are internalized via bulk endocytosis and anterogradely and retrogradely transported in neurons. J Biol Chem.

[40] Maeda S, Sahara N, Saito Y, Murayama S, Ikai A, Takashima A (2006). Increased levels of granular tau oligomers: an early sign of brain aging and Alzheimer’s disease. Neurosci Res.

[41] Maeda S, Sahara N, Saito Y, Murayama M, Yoshiike Y, Kim H, Miyasaka T, Murayama S, Ikai A, Takashima A (2007). Granular tau oligomers as intermediates of tau filaments. Biochemistry.

[42] Tang Y, Nyengaard JR, De Groot DM, Gundersen HJ (2001). Total regional and global number of synapses in the human brain neocortex. Synapse.

[43] Perez-Nievas BG, Stein TD, Tai HC, Dols-Icardo O, Scotton TC, Barroeta-Espar I, Fernandez-Carballo L, de Munain EL, Perez J, Marquie M, Serrano-Pozo A, Frosch MP, Lowe V, Parisi JE, Petersen RC, Ikonomovic MD, Lopez OL, Klunk W, Hyman BT, Gomez-Isla T (2013). Dissecting phenotypic traits linked to human resilience to Alzheimer’s pathology. Brain.

[44] Papasozomenos SC, Binder LI (1987). Phosphorylation determines two distinct species of Tau in the central nervous system. Cell Motil Cytoskeleton.

[45] Tashiro K, Hasegawa M, Ihara Y, Iwatsubo T (1997). Somatodendritic localization of phosphorylated tau in neonatal and adult rat cerebral cortex. Neuroreport.

[46] Gartner U, Janke C, Holzer M, Vanmechelen E, Arendt T (1998). Postmortem changes in the phosphorylation state of tau-protein in the rat brain. Neurobiol Aging.

[47] He HJ, Wang XS, Pan R, Wang DL, Liu MN, He RQ (2009) The proline-rich domain of tau plays a role in interactions with actin. BMC Cell Biol 10:81 10.1186/1471-2121-10-81 PMC2784441 19895707

[48] Fulga TA, Elson-Schwab I, Khurana V, Steinhilb ML, Spires TL, Hyman BT, Feany MB (2007). Abnormal bundling and accumulation of F-actin mediates tau-induced neuronal degeneration in vivo. Nat Cell Biol.

[49] Lee S, Kim W, Li Z, Hall GF (2012) Accumulation of vesicle-associated human tau in distal dendrites drives degeneration and tau secretion in an in situ cellular tauopathy model. Int J Alzheimers Dis 2012:172837 10.1155/2012/172837 PMC3270555 22315694

[50] Pooler AM, Hanger DP (2010). Functional implications of the association of tau with the plasma membrane. Biochem Soc Trans.

[51] Tolnay M, Probst A (1999). REVIEW: tau protein pathology in Alzheimer’s disease and related disorders. Neuropathol Appl Neurobiol.

[52] Stoothoff WH, Johnson GV (2005). Tau phosphorylation: physiological and pathological consequences. Biochim Biophys Acta.

[53] Iqbal K, Zaidi T, Bancher C, Grundke-Iqbal I (1994). Alzheimer paired helical filaments. Restoration of the biological activity by dephosphorylation. FEBS Lett.

[54] Dujardin S, Lecolle K, Caillierez R, Begard S, Zommer N, Lachaud C, Carrier S, Dufour N, Auregan G, Winderickx J, Hantraye P, Deglon N, Colin M, Buee L (2014) Neuron-to-neuron wild-type Tau protein transfer through a trans-synaptic mechanism: relevance to sporadic tauopathies. Acta Neuropathol Commun 2:14 10.1186/2051-5960-2-14 PMC3922636 24479894

[55] Simon D, Garcia-Garcia E, Gomez-Ramos A, Falcon-Perez JM, Diaz-Hernandez M, Hernandez F, Avila J (2012). Tau overexpression results in its secretion via membrane vesicles. Neurodegener Dis.

[56] Saman S, Kim W, Raya M, Visnick Y, Miro S, Saman S, Jackson B, McKee AC, Alvarez VE, Lee NC, Hall GF (2012). Exosome-associated tau is secreted in tauopathy models and is selectively phosphorylated in cerebrospinal fluid in early Alzheimer disease. J Biol Chem.

[57] Kfoury N, Holmes BB, Jiang H, Holtzman DM, Diamond MI (2012). Trans-cellular propagation of Tau aggregation by fibrillar species. J Biol Chem.

[58] Gendreau KL, Hall GF (2013) Tangles, Toxicity, and Tau Secretion in AD - New Approaches to a Vexing Problem. Front Neurol 4:160 10.3389/fneur.2013.00160 PMC3801151 24151487

[59] Spires-Jones TL, Stoothoff WH, de Calignon A, Jones PB, Hyman BT (2009). Tau pathophysiology in neurodegeneration: a tangled issue. Trends Neurosci.

[60] Avila J, Lucas JJ, Perez M, Hernandez F (2004). Role of tau protein in both physiological and pathological conditions. Physiol Rev.

