# Supplementary material for: Frequent and symmetric deposition of misfolded tau oligomers within presynaptic and postsynaptic terminals in Alzheimer’s disease
Source: Acta Neuropathol Commun. 2014 Oct 21;2:146. doi: 10.1186/s40478-014-0146-2 (PMC4209049; doi:10.1186/s40478-014-0146-2)
Supplement: Supplementary file 1 — Additional file 1: Figure S1: Transmission electron micrographs of human synaptoneurosomes isolated from non-demented controls, with arrowheads marking the presence of (1) myelin fragment; (2) intact synapse; (3) mitochondrion. Figure S2: Transmission electron micrographs of synaptoneurosomes isolated from Alzheimer’s disease subjects. No visible fibrils were observed. Figure S3: Immunoblotting with mouse anti-MAP2 and mouse anti-synaptophysin against total and synaptoneurosome (syn) extracts from human controls and mouse brain tissues. Low molecular weight MAP2 (<100 kDa) is highly enriched inside both human and mouse neuronal synapses. (PDF 739 KB) [file 40478_2014_9146_MOESM1_ESM.pdf]

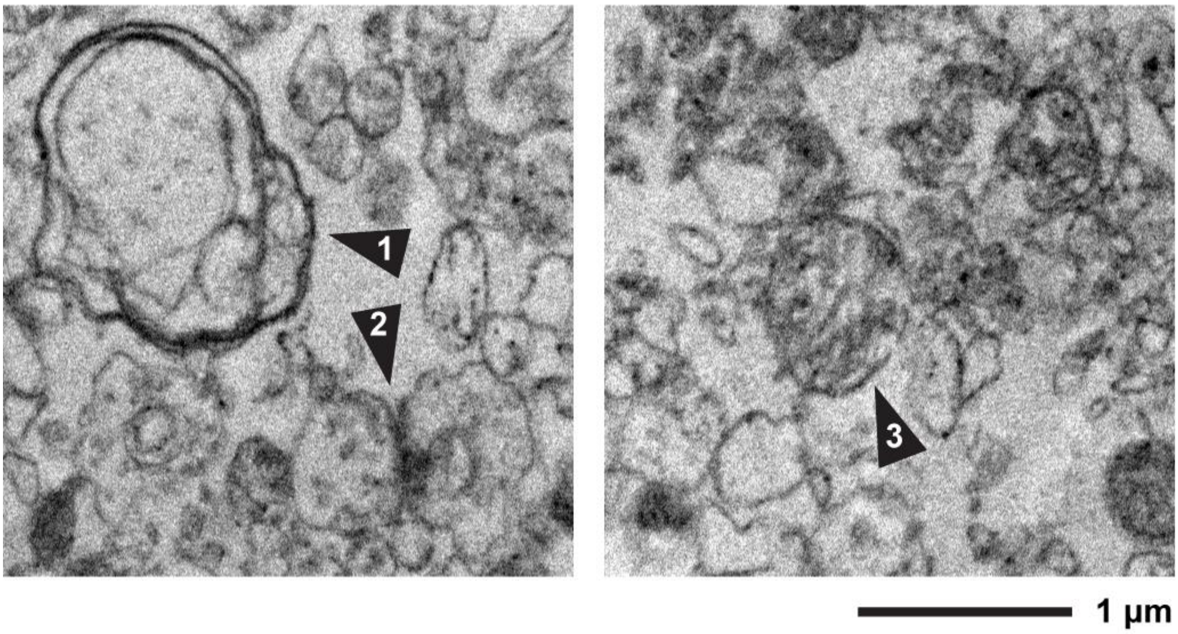

Supplementary Figure 1. Transmission electron micrographs of human synaptoneurosomes isolated from non-demented controls, with arrowheads marking the presence of (1) myelin fragment; (2) intact synapse; (3) mitochondrion.

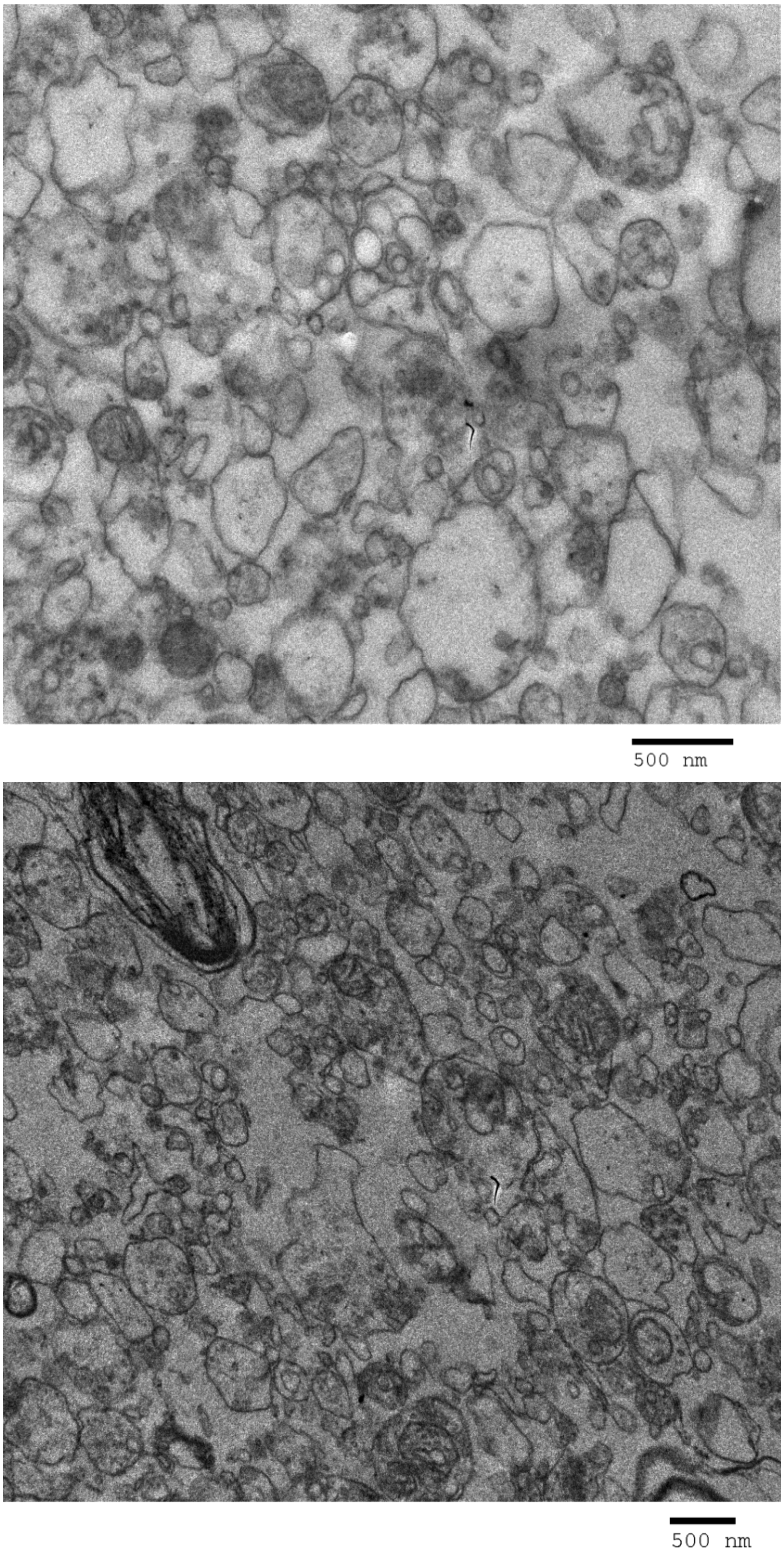

Supplementary Figure 2. Transmission electron micrographs of synaptoneurosomes isolated from Alzheimer's disease subjects. No visible fibrils were observed.

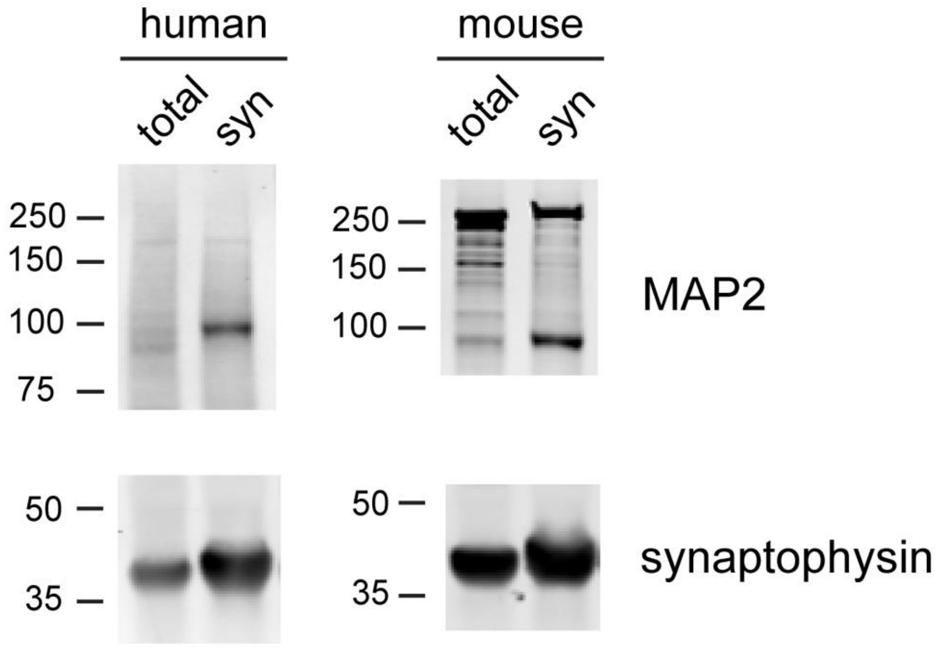

Supplementary Figure 3. Immunoblotting with mouse anti-MAP2 and mouse anti-synaptophysin against total and synaptoneurosome (syn) extracts from human controls and mouse brain tissues. Low molecular weight MAP2 (<100 kDa) is highly enriched inside both human and mouse neuronal synapses.
