# Supplementary figures and images for: Frequent and symmetric deposition of misfolded tau oligomers within presynaptic and postsynaptic terminals in Alzheimer’s disease
Source: Acta Neuropathol Commun. 2014 Oct 21;2:146. doi: 10.1186/s40478-014-0146-2 (PMC4209049; doi:10.1186/s40478-014-0146-2)

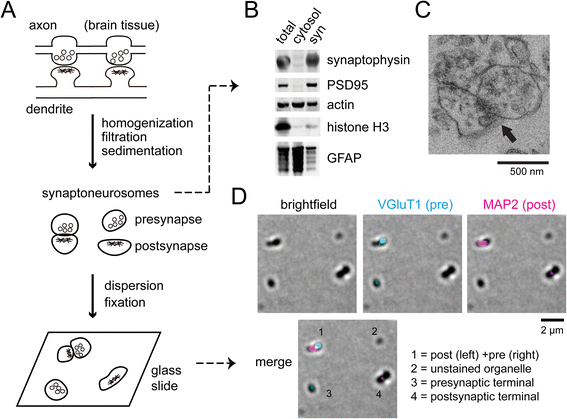

Supplement: Supplementary file 2 — Authors’ original file for figure 1 [file 40478_2014_9146_MOESM2_ESM.gif]

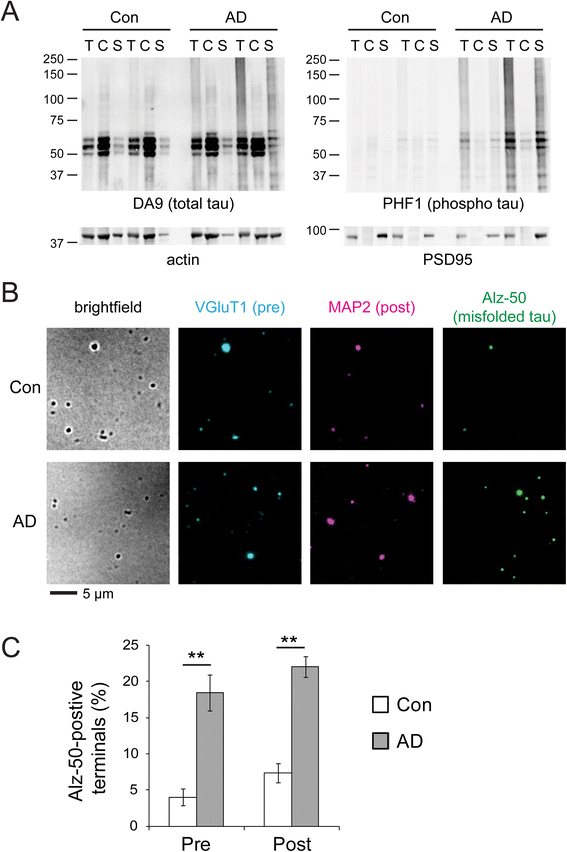

Supplement: Supplementary file 3 — Authors’ original file for figure 2 [file 40478_2014_9146_MOESM3_ESM.gif]

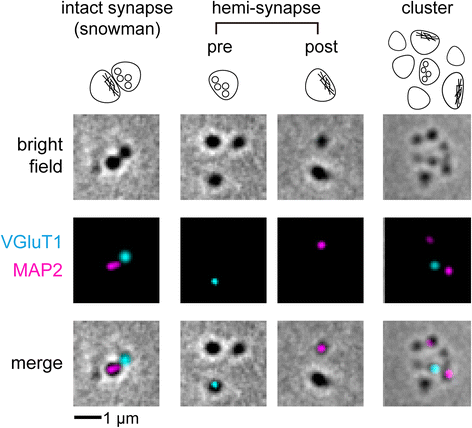

Supplement: Supplementary file 4 — Authors’ original file for figure 3 [file 40478_2014_9146_MOESM4_ESM.gif]

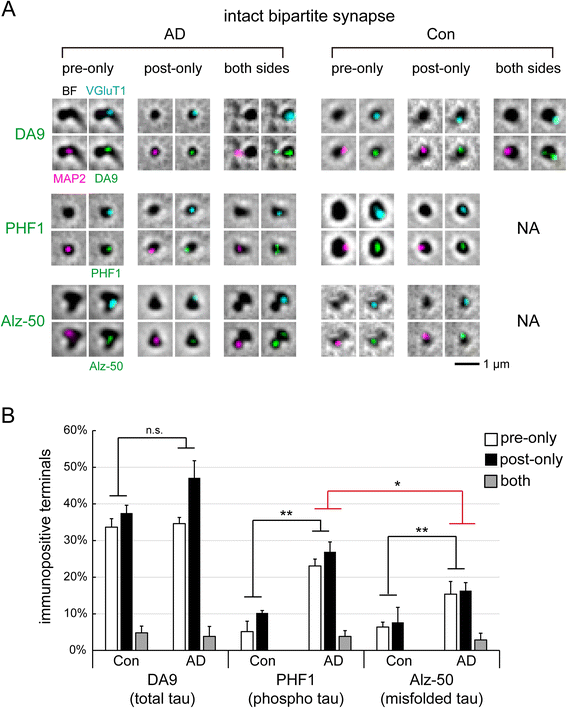

Supplement: Supplementary file 5 — Authors’ original file for figure 4 [file 40478_2014_9146_MOESM5_ESM.gif]

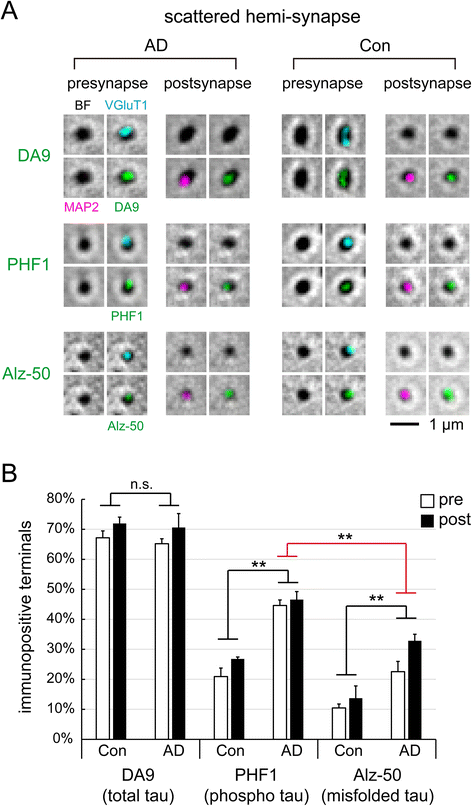

Supplement: Supplementary file 6 — Authors’ original file for figure 5 [file 40478_2014_9146_MOESM6_ESM.gif]

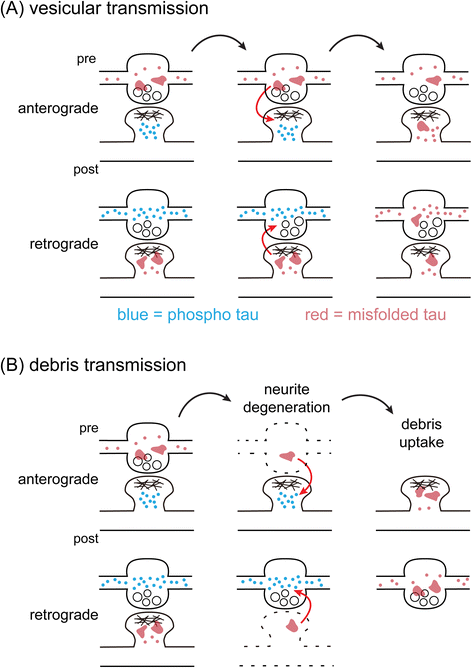

Supplement: Supplementary file 7 — Authors’ original file for figure 6 [file 40478_2014_9146_MOESM7_ESM.gif]
